# Effects of Dimethyl Anthranilate-Based Repellents on Behavior, Plumage Condition, Egg Quality, and Performance in Laying Hens

**DOI:** 10.3389/fvets.2020.00533

**Published:** 2020-08-19

**Authors:** Dušan Terčič, Mojca Pančur, Dušanka Jordan, Manja Zupan Šemrov

**Affiliations:** Department of Animal Science, Biotechnical Faculty, University of Ljubljana, Ljubljana, Slovenia

**Keywords:** poultry, feather pecking, cannibalism, beak trimming, welfare

## Abstract

Feather pecking is a behavior that occurs in order to cope with a constrained environment and is a serious problem in the egg production industry. This longitudinal study was conducted under commercial conditions to investigate whether the application of two repellent mixtures, previously suggested as aversive to wild birds, to the plumage of Prelux-R hybrid egg layers is a viable alternative to beak trimming as a solution to discourage feather pecking among laying hens. A total of 180 untrimmed hybrid layers was reared together in a floor pen. At 18 weeks of age they were allocated randomly to three treatments (repellent P, repellent T, control), each consisting of 6 replicated enriched cages with 10 hens in each cage. Hens were evenly sprayed once every 2 weeks for 54 weeks with solution P (dimethyl anthranilate and methyl phenylacetate), solution T (dimethyl anthranilate and geraniol), or distilled water (control). Body weight, plumage condition, behavior, feed intake, and egg quality measurements were taken at five time periods from 26 to 76 weeks of age. Egg production and mortality were recorded daily. The treatments did not affect feather pecking behavior. Hens treated with repellent T tended to perform less cage pecking than the control hens. The use of the repellents did not reduce feather pecking, the plumage was even more significantly damaged in the hens given the repellents compared to the control hens. This suggests the chemicals in the repellents worsened the plumage. No differences in feed intake and daily egg production between treatments were found. Raw and hard-boiled eggs were highly uniform in odor/flavor/taste and no offensive odor absorption related to the chemicals in the repellents was detected. In conclusion, in the present study we did not find any beneficial effect of dimethyl anthranilate-based repellents on feather pecking frequency and plumage/feather condition. Therefore, we do not encourage their use in wider commercial settings.

## Introduction

In modern systems for housing laying hens, producers often encounter two injurious behaviors: severe feather pecking and cannibalism, which represent a big problem from economic and animal-welfare points of view (1). Feather removal is painful for the victim (2) and the appearance of an open wound caused by the removal of feathers can lead to cannibalism, often resulting in death of the victim (3). Deterioration of feather condition also means that the energy required for body maintenance increases (4, 5). The productivity of laying hens is thus adversely affected as body maintenance must take priority over egg production. The methods most commonly used to control feather pecking are beak trimming and reducing the light intensity in the laying house (6). Although laying hens with properly trimmed beaks can cause less damage to the feathers and skin of their flock mates (7), the acute and chronic pain (8–10) that persist after the beak-trimming intervention places in jeopardy not only economic results but also the welfare of the flock (11). As a result, and also due to public concern, some European countries have introduced a ban on beak trimming while others are working toward this. Regardless of the legal prohibitions and advice of welfare spokespersons and organizations, the problem of feather pecking continues (12). It is therefore up to scientists and producers to find practical, effective, and affordable alternatives to beak trimming to accomplish similar goals. An alternative or supplement to the current approaches for controlling feather pecking and cannibalism may entail the use of effective, relatively inexpensive, easily available, non-toxic, and environmentally safe flavor chemicals that are used in human food but aversive to birds. The limited number of valuable studies available reveals that spraying feathers with repellents may help reduce the incidence of feather pecking, but the results of these studies are difficult to extrapolate to commercial practice because they were performed on experimental lines of chickens [e.g., (13, 14)], used toxic repellents, e.g., quinine [e.g., (13, 14)], or were conducted in conditions vastly different from those found on a commercial farm [e.g., (15)]. Chemical repellents like dimethyl anthranilate (DMA), methyl phenylacetate (MPA), and geraniol are already used to control avian crop depredation and nuisance problems (16–18). Although their repellent effects on wild birds have been known for many years, little has been done to develop them for practical use to improve the welfare of laying hens. Kare's report (18) mentions that DMA, MPA, and geraniol may either individually or in combinations thereof be very effective as anti-pecking agents for chickens. This record prompted us to evaluate the efficacy of two proposed repellent mixtures on the incidence and extent of feather pecking and other behaviors in a flock of commercial laying hens. The two used mixtures contained: 1) 0.78% (vol/vol) DMA and 12.50% (vol/vol) MPA and 2) 4.50% (vol/vol) DMA and 0.50% (vol/vol) geraniol which were stated as very excellent non-toxic repellent agents for prolonged periods in very small concentrations. We also aimed to investigate whether the application of repellents to hen plumage has any effect on production traits or on eggs' physical and sensory properties. To our knowledge, the repellents used in this study have not yet been used in any scientifically supported study, particularly not during the whole laying period in commercial conditions. We formulated the following hypotheses: a) dimethyl anthranilate-based repellents will deter hens from feather pecking and plumage will be less damaged in the repellent treatments than in the control treatment; b) hens in the repellent treatments will have better production results; and c) the repellents will not affect the eggs' organoleptic properties, which is vital information for both the egg processing industry and egg consumers.

## Materials and Methods

### Birds and Housing

The experiment used brown hybrid layers of the Slovenian provenance Prelux-R with intact beaks. This hybrid was chosen for two reasons. First, several studies in the past showed that hens with brown feathers are more prone to feather pecking than those with white feathers (19). It is also long known that hens' tendency to feather peck depends on genetics (20, 21) and our preliminary studies showed that, under the same environmental conditions and among the several genotypes tested, the Prelux-R strain was the most prone to feather pecking behavior. Throughout the experiment, the birds were kept in a ventilated windowless poultry house. After hatching, the chicks were housed in a deep litter system on the farm at the Biotechnical Faculty University of Ljubljana. From hatching to 17 weeks (wk) of age, the chicks and later the pullets were exposed to a typical lighting program, meaning they had one period of light and one period of darkness within 24 h. During the first week, fluorescent lights were on for 23 h, while for 1 h the birds were in the dark. After that, the lighting was gradually reduced by 2.5 h per week until it reached 8 h a day in wk 7, which was then maintained until wk 17. In the first week of age, the air temperature at the chick level ranged between 32 and 35°C and was then lowered by about 2.5°C every week so that at wk 7 a temperature of 20°C was reached, which is also the optimal temperature for adult hens. Two balanced feed mixtures, a starter (11.9 MJ ME/kg, 20% CP) from hatching to wk 10 and a developer (11.4 MJ ME/kg, 15% CP) from wk 11 to wk 18, were offered to the birds during the rearing period. Both feed and drinking water were provided on an *ad libitum* basis. A total of 180 pullets were transferred to enriched cages (Officine Facco & C. Spa, Italy) of a three-tier system at the age of 18 wk. The dimensions of each cage were 116 cm (length) × 62 cm (width). The cages were equipped with nests (730 cm^2^/cage), round metal perches (15 cm/hen), scratch plates, and claw-shortening devices. The minimum height of the cage above the usable and non-usable area was 45 cm. Cages provided 646 cm^2^ of usable space area per hen. At the front of the cages there was a feeder trough and on the back there were four nipples. Ten hens were placed in each cage. The duration of exposure to light was gradually increased from 8 h of light at wk 17 to 15 h light at wk 24 and onwards. The light intensity measured at the feed trough of the middle tier was maintained at around 15 lux. From wk 18 to wk 76, the laying hens were fed a complete layer feed (11.3 MJ ME/kg, 16.2% CP) in crumble form. Laying hens were aged 76 wk when the trial was terminated.

### Treatment Groups, Repellent Applications, and Data Collection

**Figure 1:**
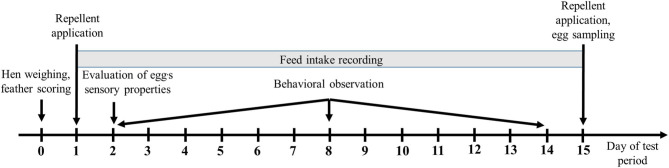
Tasks completed during each day of test period.

Hens were tagged with leg rings and randomly divided into 3 treatments of 60 hens each when they were transferred from the deep litter system to the enriched cages. Each cage with 10 hens represented 1 replication, and 6 cages were allocated to each treatment. In each treatment, care was taken to ensure that the hens were equally located in cages on the upper, middle, and bottom battery levels. A physical separation (empty cages) between the three treatment groups was introduced to prevent cross-contamination while applying the repellent. Two repellent solutions were prepared: solution P and solution T. The composition of solutions P and T is shown in Table 1. Distilled water was applied to the plumage of hens in the control group. All chemicals used were obtained from the Sigma-Aldrich company (Sigma-Aldrich Chemie, Germany). The repellents/distilled water were applied every 14 days at the same time. This time frame was decided based on Kare's (18) claim that the repellents used provide a deterrent effect for several weeks and also so as to take logistical and economic factors into account. A total of 28 applications was performed, the first at wk 20 and the last at wk 74. Sprayers powered by compressed air from an air compressor and equipped with metal nozzles were used to apply the repellents/distilled water. Throughout the experiment, the same three sprayers were used, one for each treatment group. After each use, they were thoroughly cleaned with a brush, washed and rinsed with clean water. The same operator sprayed the birds evenly and repeatedly. In a separate, pretreatment study, we determined that 300 ml of repellent/distilled water was sufficient to thoroughly wet the plumage of hens in each treatment. With each application of repellent mixtures, the P treatment group received a dose of ~39 μl DMA and 625 μl MPA per hen, while the T treatment group received a dose of ~225 μl DMA and 25 μl geraniol per hen. Egg production per cage and mortality per cage were monitored daily and throughout the production period. All other production and behavioral data were collected over five 2-week test periods spread over the entire production cycle. In these periods, the age of the hens was as follows: wk 26–28 (period 1), wk 38–40 (period 2), wk 50–52 (period 3), wk 62–64 (period 4) and wk 74–76 (period 5). Each of the five bi-weekly test periods began 1 day before the repellents/distilled water were applied and ended with the next application. A more detailed sequence of the observations, evaluations, and measurements conducted within each test period is shown in Figure 1. The first weighing and evaluation of feather condition was performed when the hens were 20 wk old and later regularly at the start of each period. Therefore, six weighings and assessments of the feather condition were performed. Feed intake was measured on a daily basis prior to the morning feeding by weighing the feed remaining in the trough. Feed intake was then expressed as average daily intake per hen. Temperature and relative humidity inside the house were recorded continuously using Data logger 42270 (Extech Instruments, Townsend West Nashua, NH, USA), which was located at the height of the middle battery level and at a distance of 0.9 m away from the battery.

**Table 1:** Chemical composition of the repellents used.

**Treatment**	**Volume concentration (%)**	**Ingredient (molecular formula)**	**CAS^a^ number**	**Color and form**
Repellent P	0.78	Dimethyl anthranilate (C_9_H_11_NO_2_)	85-91-6	Colorless to yellow liquid
	12.50	Methyl phenylacetate (C_9_H_10_O_2_)	101-41-7	Colorless liquid
	86.72	Propylene glycol (C_3_H_8_O_2_)	57-55-6	Colorless liquid
Control	100.00	Distilled water (H_2_O)	7732-18-5	Colorless liquid
Repellent T	4.50	Dimethyl anthranilate (C_9_H_11_NO_2_)	85-91-6	Colorless to yellow liquid
	0.50	Geraniol (C_10_H_18_O)	106-24-1	Colorless to pale yellow oily liquid
	5.00	Polysorbate 80 (C_64_H_124_O_26_)	9005-65-6	Yellow liquid
	90.00	Distilled water (H_2_O)	7732-18-5	Colorless liquid

a *Chemical Abstracts Service (CAS) registry number*.

### Behavior and Plumage Condition

Within each of five bi-weekly periods, behavior was observed for 3 days (days 2, 8, and 14; Figure 1). Each day, data were collected in three sessions; morning (9:00 a.m. to 10:30 a.m.), afternoon (12:15 p.m. to 1:45 p.m.) and evening (3:30 p.m. to 5:00 p.m.). The behavior of the hens was monitored by direct observation such that the hens in each cage were observed once in each session. Thus, 5 min was available for each cage. Within this 5-min period, 2 min was allotted for the observer to move to the next cage and for the hens to become acclimatized to their presence while the behavior was observed for the next 3 min. These 3 min of observation were subdivided into 15-second intervals making a total of 12 intervals per session, altogether giving 9,720 sample points throughout the experiment (5 test periods × 3 observation days/test period × 3 sessions/observation day × 12 sample points/session × 18 cages). Nine behaviors observed in three daily sessions are listed in Table 2. Feeding behavior was scored for 15-second intervals using scan sampling while for other behaviors one-zero sampling within these intervals was performed. An interval was marked as 1 if at least one bird was performing the behavior, or 0 if not. All behaviors except for drinking were scored in the front part of the cage (up until the perch line) because it was harder to see the birds in the rear part. During observations, the observer stood ~0.6 m away from the front side of the cages. This position permitted an unobstructed view of all hens in a particular cage, without the disturbance of any hen. All observations were made by the same trained and experienced observer. Tauson et al.'s scoring method (22) was used to assess the condition of plumage. Briefly, each hen from each cage was first weighed and then given a score for its plumage condition on a scale from 1 (worst plumage condition) to 4 (best plumage condition). Each hen was then scored for six individual body parts (back, wings, tail, cloaca/vent region, neck, breast). In addition to this method, images provided by Tauson et al. (22) for brown genotypes illustrating the scoring scale were used while making the plumage assessments. In order to avoid individual observer bias, the assessment of plumage condition was always carried out by two trained persons, and the same two persons gave the scores in each test period. The plumage condition assessment was done at the same time by both assessors and one consent score per body area was given. All scores were added for a total plumage score.

**Table 2:** Ethogram of the caged hens.

**Behavior**	**Description**
Feeding^D^	Pecking of feed in the feeding trough and picking up feed particles from the edge of the feeding trough
Drinking^F^	Pecking of the nipples and soaking the beak in the groove underneath the nipples
Head-directed pecking^F^	Any pecking of the head, except pecking of the beak of another hen
Continuous feather pecking^F^	Pecking at the feathers of another hen when the hen pecks the other hen at least twice in a row
Individual feather pecking^F^	Pecking at the feathers of another hen when the hen pecks the other hen exactly once.
Preening^F^	Using the beak to clean the feathers
Comfort activity^F^	Shaking of the whole body during which the hen's feathers are in ruffled position and wings flap
Cage pecking^F^	Pecking of all parts of the cage, except for the feeding trough; cage pecking also involves approaching a cage structure with a beak
Pecking in the air^F^	Pecking is directed into an empty space and includes the pecking of dust particles in the air

*D, Long-term behavior evaluated as duration; F, short-term behavior evaluated as frequency*.

### Assessments of Physical and Sensory Characteristics of the Eggs

At the end of each test period (day 15—Figure 1), a random sample of six freshly laid eggs per cage was selected to assess physical quality parameters. To ensure lay time uniformity, eggs were gathered by hand from the cage row within 4 h of being laid, placed in cardboard egg trays, and shipped to the laboratory. For each test period, 108 eggs were utilized, comprising 36 eggs for each of the three different treatments. A total of 540 eggs was used in the whole study. Egg quality measurements were tracked for individual eggs. Egg weight, shell color, albumen height, yolk color and Haugh units were recorded with the QCM+ System (Technical Services and Supplies, York, UK). The height of the inner thick albumen was measured in a position ~1 cm away from the edge of the yolk. The Haugh units were calculated by converting egg weight and albumen height values as follows (23): Haugh unit = 100 log (H – 1.7W^0·37^+ 7.6), where H is the albumen height expressed in millimeters and W is the weight of an egg expressed in grams. The height and width of an egg as well as the diameter of the yolk were measured using a digital caliper. Shell strength was tested with an Instron 3342 device (Instron Ltd, Norwood, MA, USA) equipped with a 50 N load cell and 5 cm diameter compressions disc. While measuring shell compression strength, an egg was placed on its side (horizontal position) in a metal egg holder. The volumes of thick and thin albumen were mixed with a spatula before measuring the pH value. Albumen and yolk pH were measured with a S47-K Seven Multi dual pH/conductivity meter (Mettler-Toledo, Schwerzenbach, Switzerland). All egg analyses were performed in a laboratory at room temperature. Due to the lack of analytical techniques, possible residues of the repellents in the eggs could not be determined. An egg is a food that can absorb foreign odors from the environment and these foreign odors can produce unpleasant odors and tastes in an egg. Three organic compounds with medium to strong odor were used in the experiment. The odors of these chemicals can be described by humans as orange-blossom and grape-like (DMA), strong, very sweet, like honey (MPA) and floral, and rosey (geraniol). To determine whether the application of either repellent solution affects the sensory properties of the eggs, especially their odor, flavor, and taste, 20 eggs from each of the three treatments were taken for sensory analyses. Egg samples were taken 1 day after application of the repellents. All eggs were carefully weighed to ensure they had the same weight. Sensory analyses were performed with four volunteer subjects. During the preliminary tests, volunteers were familiarized with the sensory evaluation procedure. After each test of a hard-boiled egg, the volunteers rinsed their mouths out with water, then rested for up to 2 min before evaluating the next sample. Immediately before the sensory evaluations, the eggs had been cooked on a low heat for 9 min. After 9 min, they were taken out of the water, cooled for 2 to 3 min under running cold water and then peeled. For all eggs, the temperature in the center of the egg was approximately the same, between 90 and 93°C. From each treatment group, three cooled, hard-boiled, peeled, and blind coded eggs as well as two blind coded raw eggs were offered in a randomized design to each volunteer for sensory evaluation. The reference sample was eggs from the control group. Samples of eggs were placed before each volunteer on plastic plates under a white fluorescent light. The eggs' sensory properties were evaluated using a descriptive analytical test to quantify individual sensory properties. An unstructured 7-point scale was used where 1 point means that the property is not expressed or is totally unacceptable, and 7 points that the property is strongly or perfectly expressed. Volunteers were first asked to evaluate the odor, the presence of any foreign odors, the shape and consistency of the yolk, and the consistency of the albumen of the raw eggs. In the second step, they evaluated the odor, the presence of any foreign odors, the texture (feeling in the mouth), the taste, the aftertaste, and the overall impression of the hard-boiled eggs.

### Statistical Analyses

The analyses were conducted using SAS software (SAS Institute Inc., Proprietary Software 9.4, Cary, NC, USA, 2016). Distributions of the means and residuals were examined to check the model assumptions. Statements of statistical significance were based on *P* < 0.05. Feather scores were analyzed using the PROC GENMOD procedure with a Poisson regression, as is often used for categorical variables that only take a limited number of discrete values. Treatment and test period represented the fixed factors in the analysis, while plumage condition of the back, area around the cloaca/vent, sum of the back and cloaca, and plumage condition expressed as the sum of all body part scores were dependent variables. As a covariate, the body weight of the hens was incorporated into the model. Hen within cage was used in a repeated statement. Feeding behavior as the sum of duration per cage and the period in seconds as well as the frequency of short-term behaviors were analyzed using the PROC MIXED procedure with the test period, treatment, observation day within test period, and their interactions as the categorical variables. The effects of the observation day and interactions were not significant (P>0.05), and were therefore dropped from the final statistical models. The short-term behaviors were expressed as proportions of 9,720 sample points during which the behaviors occurred. Egg quality characteristics and feed intake data were analyzed using the PROC MIXED procedure with the test period, treatment, and their interaction as the categorical variables. In the statistical models for behaviors, egg quality, and feed intake, the effect of cage was modeled as a random effect. For multiple comparisons, the Tukey-Kramer method was used. There was strong evidence of a treatment by period interaction for albumen pH [*F*_(8, 501)_ = 2.20, *P* = 0.03] and yolk pH [*F*_(8, 500)_ = 6.04, *P* < 0.0001], but due to its complexity they are not presented here. The values for the eggs' sensory properties were not normally distributed and scoring was limited to seven discrete values. The NPAR1WAY procedure (non-parametric Wilcoxon test) was used to statistically evaluate this data. Hen-day egg production data (the percentage of hens in lay corrected for mortality) did not meet the assumption of the parametric statistical tests and were therefore subjected to an arcsin transformation. After performing the statistical test on the transformed data, a back transformation of the results was performed to obtain more informative values. Mortality data were analyzed using a chi-square test.

## Results

### Behavior and Plumage Condition

**Figure 2:**
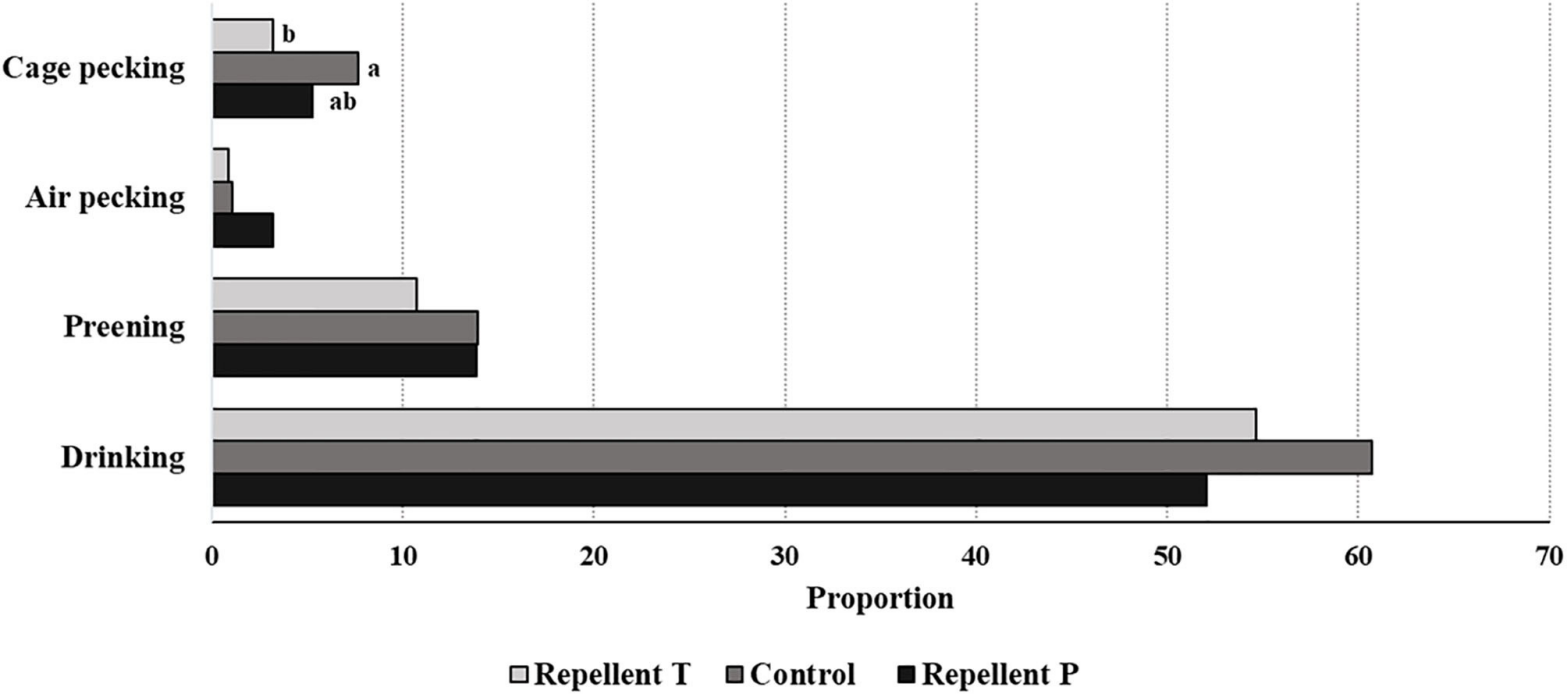
Effect of the treatment on short-term behavior (least square means of the same behavior lacking a common letter differ— *P* < 0.07).

**Figure 3:**
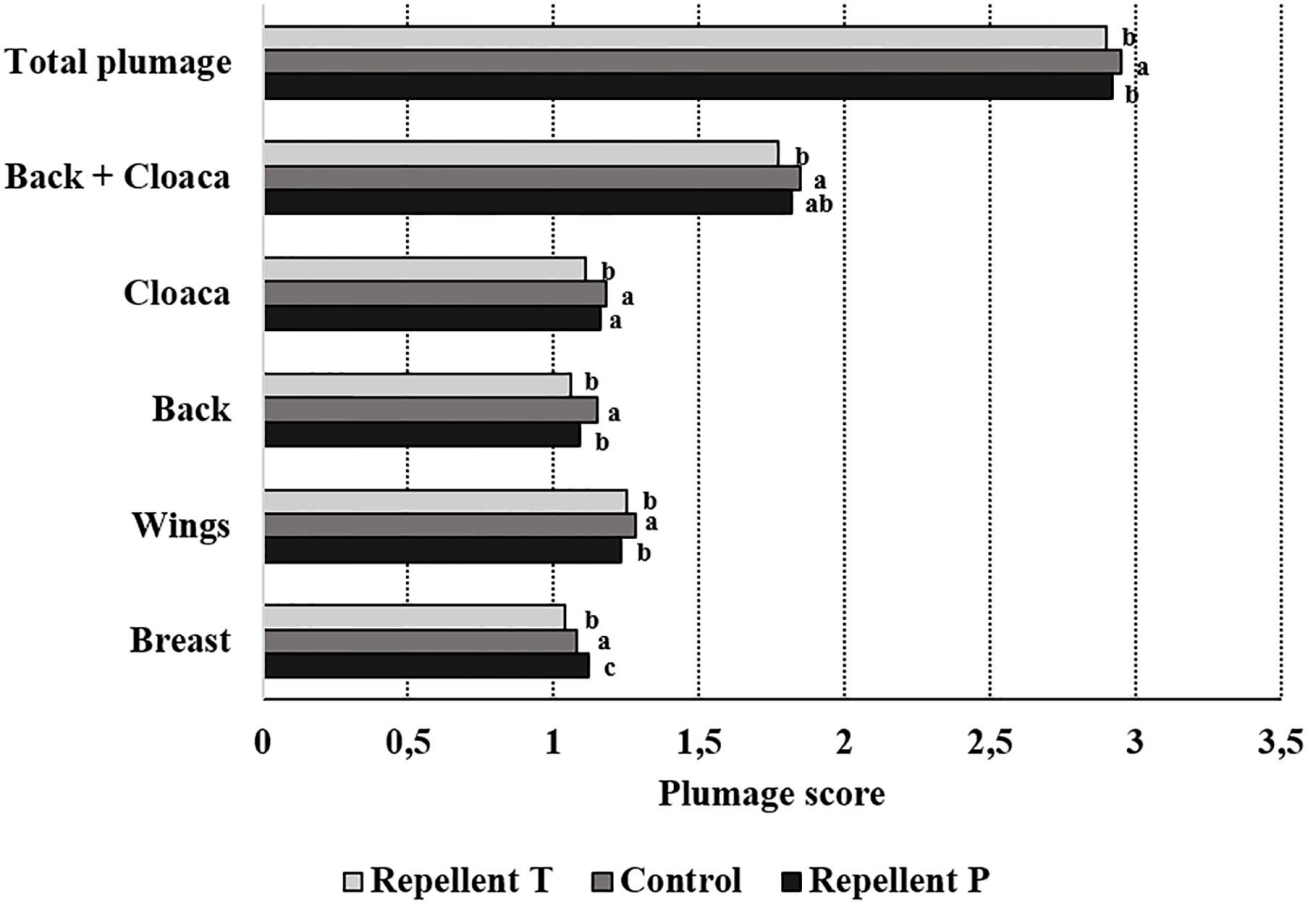
Effect of the treatment on plumage condition (means of the same body part lacking a common letter differ— *P* < 0.05).

Spraying hens' feathers with repellents had no effect on hen behavior. The only effect of repellent treatment found was for cage pecking behavior [*F*_2, 59_ = 2.64; *P* = 0.08] (Figure 2). Control birds tended to show more cage pecking than birds treated with repellent T (estimate = 4.23 ± 1.88; *P* = 0.06). Regardless of the treatment, the birds performed with similar frequency head-directed pecking [*F*_2, 60_ = 0.08; *P* = 0.92], feather pecking [individual and continuous pecking combined; *F*_2, 60_ = 0.09; *P* = 0.90], drinking [*F*_2, 60_ = 1.42; *P* = 0.25], preening [*F*_2, 60_ = 0.38; *P* = 0.69], air pecking [*F*_2, 60_ = 0.71; *P* = 0.49], comfort behavior [*F*_2, 60_ = 1.25; *P* = 0.29] and peck-related behaviors (all recorded behaviors except feeding and comfort activity; *F*_2, 60_ = 0.76; *P* = 0.47). Birds also showed similarly long feeding behavior (LSM ± SE in seconds; *P* = 1146.50 ± 57.53, T = 1182.50 ± 57.53, Control = 1205.00 ± 60.00; *F*_2, 60_ = 0.26; *P* = 0.77). Considering the effect of test period, the behavior was different with feeding duration getting shorter with each subsequent test period [*F*_4, 60_ = 11.06 *P* < 0.0001]. The frequency of head-directed pecking [*F*_4, 60_ = 5.12; *P* = 0.001] as well as drinking [*F*_4, 60_ = 19.75; *P* < 0.0001] decreased with age (test period). Cage pecking did not show any particular pattern with time [*F*_4, 59_ = 4.09; *P* = 0.005] while comfort activity [*F*_4, 60_ = 11.33; *P* < 0.0001] and preening [*F*_4, 60_ = 0.49; *P* = 0.0006] were gradually less performed with a small peak in the last period. Peck-related behaviors were at their highest in the first two periods then they decreased, only to rise again in the last period [*F*_4, 60_ = 11.33; *P* < 0.0001]. No effect of test period was found for feather pecking [*F*_4, 60_ = 0.25; *P* = 0.91] and air pecking [*F*_4, 60_ = 1.20; *P* = 0.31]. Observation day within the test period had no effect on any of the observed behavior. Plumage condition was worse in hens from both repellent-treated groups compared to the hens in the control group (total plumage; Chi Square = 12.04, *P* = 0.002). Hens from the control group showed better plumage condition on the wings (Chi Square = 12.90, *P* = 0.002) and back (Chi Square = 9.54, *P* = 0.009). Hens from the T group had worse plumage on the breasts (Chi Square = 26.22, *P* < 0.0001) and around the cloaca compared to the other birds (Chi Square = 8.49, *P* = 0.014) and worse on the back compared to the hens in the control group (Chi Square = 10.88, *P* < 0.0001) (Figure 3). The test period had an effect on plumage condition on each body part investigated and deteriorated with each subsequent test period (Table 3; total plumage, Chi Square = 158.25; *P* < 0.0001).

**Table 3:** Effect of the test period on plumage condition.

**Test period (age of hens)**	**Breast**	**Wings**	**Back**	**Cloaca**	**Back + Cloaca**	**Total plumage^1^**
Before the trial (wk 20)	1.42^a^ ± 0.010	1.39^a^ ± 0.010	1.41^a^ ± 0.012	1.42^a^ ± 0.006	2.10^a^ ± 0.017	3.20^a^ ± 0.010
1st (wk 26)	1.39^b^ ± 0.002	1.38^a^ ± 0.002	1.37^a^ ± 0.002	1.39^a^ ± 0.013	2.07^b^ ± 0.003	3.17^b^ ± 0.002
2nd (wk 38)	1.24^c^ ± 0.013	1.37^b^ ± 0.004	1.22^b^ ± 0.014	1.32^b^ ± 0.011	1.96^c^ ± 0.010	3.06^c^ ± 0.006
3rd (wk 50)	0.99^d^ ± 0.014	1.26^c^ ± 0.011	0.99^c^ ± 0.003	1.13^c^ ± 0.005	1.75^d^ ± 0.021	2.84^d^ ± 0.012
4th (wk 62)	0.81^e^ ± 0.017	1.12^d^ ± 0.018	0.84^d^ ± 0.010	0.99^d^ ± 0.002	1.61^e^ ± 0.024	2.71^e^ ± 0.015
5th (wk 74)	0.61^f^ ± 0.016	0.99^e^ ± 0.017	0.78^e^ ± 0.004	0.66^e^ ± 0.004	1.41^f^ ± 0.030	2.58^f^ ± 0.016

*Results are presented as least squares means (LSM) ± SE*.

a, b, c, d, e, f *Means within a column of the same body part lacking a common superscript differ (P < 0.05)*.

1 *Refers to the sum of scores for all six body parts*.

### Performance

Regardless of the treatment, feed intake [*F*_2, 15_ = 0.44; *P* = 0.65] and hen-day egg production [*F*_(2, 7056)_ = 0.18; *P* = 0.84] were similar during the production period. A statistical analysis performed on feed intake data revealed a significant main effect of test period [*F*_(4, 1229)_ = 12.33; *P* < 0.0001] and a test period × treatment interaction [*F*_(8, 1229)_ = 2.96; *P* = 0.003]. The interaction test period × treatment was considered complex and not informative as it was hard to discern a general pattern and it was not possible to discuss trends for the main effect of one factor for each level of the other factor. Mortality was not different between treatments (Chi Square = 0.33, *P* = 0.56) and, according to our examination of dead birds, was not cannibalism-associated (Table 4).

**Table 4:** Production parameters of laying hens displayed by test periods and treatment.

	**Feed intake (g per hen per day)**						**Hen-day egg production (%)**	**Mortality (%)**
**Test period (age of hens)**	**1st (wk 26–28)**	**2nd (wk 38–40)**	**3rd (wk 50–52)**	**4th (wk 62–64)**	**5th (wk 74–76)**	**Entire trial (wk 26–76)**	**Entire trial (wk 26–76)**	**Entire trial (wk 26–76)**
Control group	128.12^a^^B^ ± 4.62	132.27^a^^AC^ ± 4.62	135.78^a^^A^ ± 4.62	129.80^a^^BC^ ± 4.63	131.59^a^^BC^ ± 4.62	131.51^a^^A^ ± 4.44	82.47^a^ ± 1.42	11.7^a^
Repellent T	137.80^a^^A^ ± 4.62	138.69^a^^A^ ± 4.62	138.64^a^^A^ ± 4.62	131.18^a^^B^± 4.62	139.14^a^^A^ ± 4.62	137.09^a^^A^ ± 4.44	81.90^a^ ± 1.42	11.7^a^
Repellent P	131.14^a^^AB^ ± 4.62	139.29^a^^C^ ± 4.62	133.26^a^^B^ ± 4.62	127.52^a^^A^ ± 4.62	132.37^a^^B^± 4.62	132.72^a^^A^ ± 4.44	81.26^a^ ± 1.42	1.6^a^

*Values are expressed as least squares means (LSM) ± SEM*.

a *means within a column of the same production parameter lacking a common superscript differ (P < 0.05)*.

A, B, C *means within a row of the same treatment lacking a common superscript differ (P < 0.05)*.

### Physical Characteristics of the Eggs

**Figure 4:**
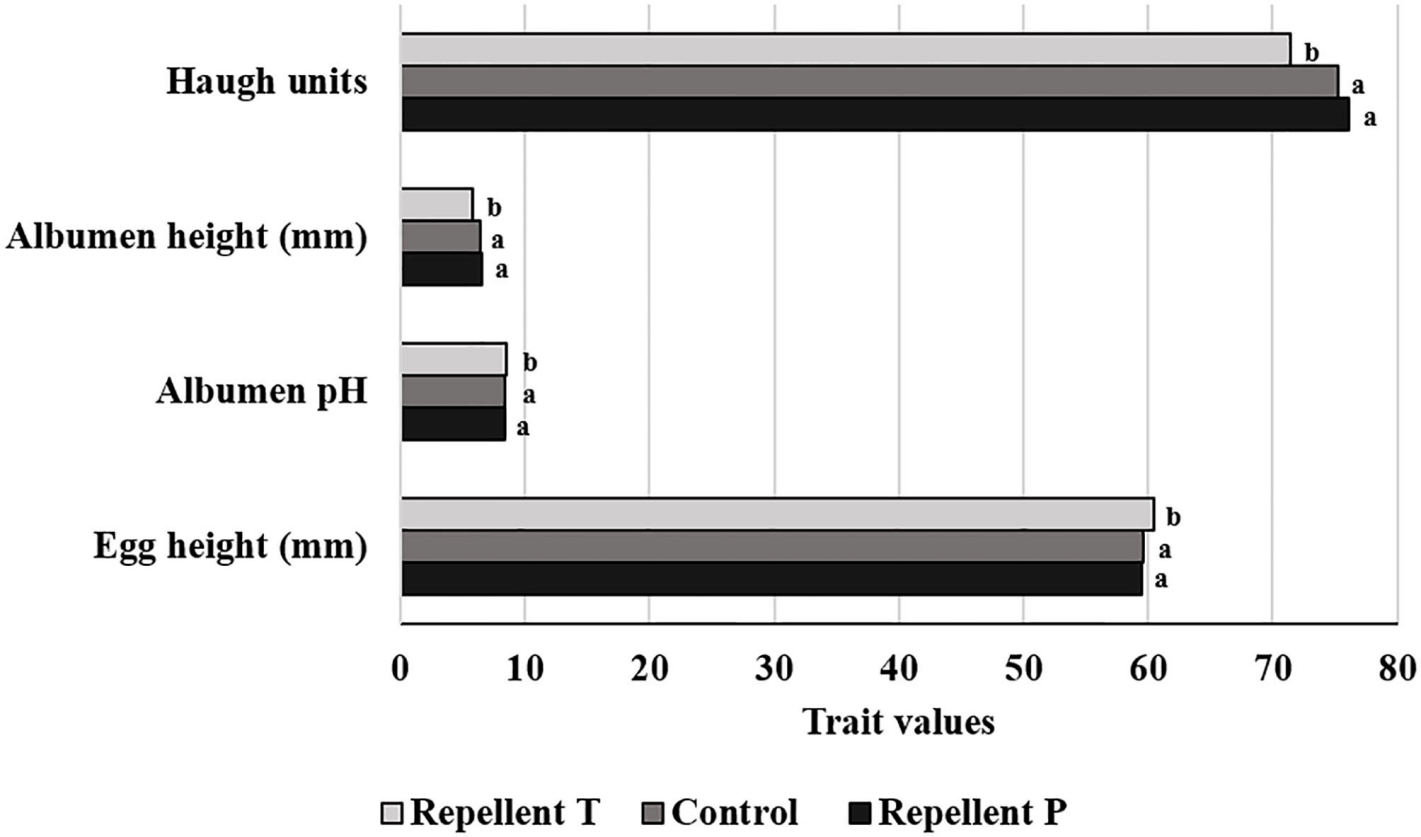
Effect of the treatment on four egg physical characteristics (means of the same egg trait lacking a common letter differ— *P* < 0.05).

Analysis of the egg quality data revealed a difference (*P* < 0.001) between treatments in the following egg quality traits: egg height, albumen pH, albumen height, and Haugh units (Figure 4). Hens treated with repellent T laid eggs with a greater height than did other hens [*F*_(2, 505)_ = 10.51, *P* < 0.0001]. Considering all treatments, the highest deterioration of egg quality as measured by albumen pH [*F*_(2, 501)_ = 19.78, *P* < 0.0001], albumen height [*F*_(2, 504)_ = 11.97, *P* < 0.0001], and Haugh units (*F*_(2, 495)_ = 8.34, *P* = 0.0003] was seen for the repellent T treatment. Hens undergoing the repellent T treatment produced eggs with a higher albumen pH (*P* < 0.0001) and a lower albumen height (*P* < 0.001) and Haugh units (*P* < 0.001) than those from the repellent P and control treatments (Figure 4). There was no difference between the repellent P group and the control group in least square mean values of egg height, albumen pH, albumen height, and Haugh units. The effects of test period on the exterior and interior egg quality indicators are presented in Table 5. The results of the study showed the effect of test period (hen age) on all recorded egg physical characteristics, egg weight [*F*_(4, 506)_ = 37.37, *P* < 0.0001], egg width [*F*_(4, 505)_ = 11.40, *P* < 0.0001], egg height [*F*_(4, 505)_ = 70.23, *P* < 0.0001], shell color [*F*_(4, 505)_ = 14.19, *P* < 0.0001], shell strength [*F*_(4, 505)_ = 29.19, *P* < 0.0001], yolk diameter [*F*_(4, 495)_ = 153.07, *P* < 0.0001], albumen pH [*F*_(4, 501)_ = 6.72, *P* < 0.0001], yolk pH [*F*_(4, 500)_ = 48.65, *P* < 0.0001], yolk color [*F*_(4, 501)_ = 2.70, *P* = 0.0302], albumen height [*F*_(4, 504)_ = 51.20, *P* < 0.0001], and Haugh units [*F*_(4, 495)_ = 58.04, *P* < 0.0001]. Shell strength, albumen height, and Haugh units decreased (*P* < 0.001) throughout the experimental period with the highest values (43.75 N, 7.53 mm, 85.17, respectively) occurring at the age of 28 wk and lowest values (32.77 N, 5.39 mm, 66.94, respectively) at the age of 76 wk (Table 5). Conversely, egg weight, egg width, and egg height increased as the hen age increased. The weight of an egg ranged from 63.15 g initially to 69.91 g at the end of the trial (Table 5). The eggshell was brighter (*P* < 0.05) in the second and third test periods than in the first and fifth periods, while the yolk was brighter in the fourth than in the first and the second periods. Albumen and yolk pH were decreasing until the fourth test period, but then increased again in the last test period.

**Table 5:** Effect of the test period on egg physical characteristics.

**Test period (age of hens^1^)**	**1st (wk 28)**	**2nd (wk 40)**	**3rd (wk 52)**	**4th (wk 64)**	**5th (wk 76)**
Egg weight (g)	63.15^d^ ± 0.37	66.35^a^ ± 0.52	67.64^ab^ ± 0.52	69.11^bc^ ± 0.52	69.91^c^± 0.53
Egg width (mm)	44.12^a^ ± 0.09	44.56^ab^ ± 0.13	44.75^bc^ ± 0.13	44.63^b^ ± 0.13	45.17^c^ ± 0.13
Egg height (mm)	57.59^b^ ± 0.16	58.89^b^ ± 0.22	60.03^b^ ± 0.22	61.17^a^ ± 0.22	61.46^a^ ± 0.22
Shell color (%)	34.86^b^ ± 0.42	39.65^a^ ± 0.60	38.91^a^ ± 0.60	37.50^ac^ ± 0.60	36.18^bc^ ± 0.60
Shell strength (N)	43.75^a^ ± 0.63	41.18^ab^ ± 0.88	38.59^bc^ ± 0.88	36.66^c^ ± 0.88	32.77^d^ ± 0.89
Yolk diameter (mm)	39.46^c^ ± 0.13	43.32^ab^ ± 0.18	43.78^a^ ± 0.18	43.71^a^ ± 0.18	42.64^b^ ± 0.18
Albumen pH	8.47^b^ ± 0.01	8.39^ab^ ± 0.02	8.41^ab^ ± 0.02	8.32^a^ ± 0.02	8.46^b^ ± 0.02
Yolk pH	6.19^c^ ± 0.01	6.13^a^ ± 0.01	6.07^ab^ ± 0.01	6.03^b^ ± 0.01	6.30^c^ ± 0.01
Yolk color (Roche)	11.08^a^ ± 0.04	11.04^a^ ± 0.05	11.08^ab^ ± 0.05	11.29^b^ ± 0.06	11.10^ab^ ± 0.06
Albumen height (mm)	7.53^c^ ± 0.10	6.35^a^ ± 0.14	6.33^a^ ± 0.14	5.64^b^ ± 0.14	5.39^b^ ± 0.14
Haugh units	85.17^c^ ± 0.81	75.27^a^ ± 1.15	75.29^a^ ± 1.16	68.50^b^ ± 1.14	66.94^b^ ± 1.16

*Values are expressed as least squares means (LSM) ± SEM*.

a, b, c, d *different superscripts on LSM values within a row indicate a difference (P < 0.05)*.

1 *Age of the hens in weeks (wk) at the time of egg sampling*.

### Sensory Properties of the Eggs

The sensory panelists did not find any difference (*P* < 0.05) between treatments in the sensory properties of the raw egg and hard-boiled egg samples (Table 6). The majority of sensory properties were scored higher than 6.0. One exception was the consistency of the albumen in raw eggs, where eggs were given scores of < 6. In all three treatments, a small number of eggs was found to have an aftertaste (hard, metallic). The coefficients of variation indicated that, for this trait, the range of variability relative to the mean value was also the greatest (Table 6).

**Table 6:** Sensory quality ratings of raw and hard-boiled eggs by treatment.

**Sensory properties**	***P*-value**	**Control group**		**Repellent T**		**Repellent P**	
		**$\bar{X}\pm \sigma$**	**CV (%)**	**$\bar{X}\pm \sigma$**	**CV (%)**	**$\bar{X}\pm \sigma$**	**CV (%)**
**Raw eggs**							
Odor (1–7)^a^	0.5234	6.95 ± 0.22	3.17	6.93 ± 0.23	3.32	6.87 ± 0.19	2.77
Foreign odors (1–7)^a^	1.0000	1.00 ± 0.00	0.00	1.00 ± 0.00	0.00	1.00 ± 0.00	0.00
Shape of yolk (1–7)^a^	0.4825	6.42 ± 0.43	6.70	6.38 ± 0.54	8.46	6.51 ± 0.43	6.61
Consistency of yolk (1–7)^a^	0.8357	6.44 ± 0.48	7.45	6.38 ± 0.52	8.15	6.51 ± 0.47	7.22
Consistency of albumen (1–7)^a^	0.7942	5.89 ± 0.63	10.70	5.84 ± 0.76	13.01	5.91 ± 0.81	13.71
**Hard-boiled eggs**							
Odor (1–7)^a^	0.5932	6.14 ± 0.41	6.68	6.12 ± 0.49	8.01	6.22 ± 0.39	6.27
Foreign odors (1–7)^a^	1.0000	1.00 ± 0.00	0.00	1.00 ± 0.00	0.00	1.00 ± 0.00	0.00
Texture (1–7)^a^	0.1826	6.18 ± 0.41	6.63	6.11 ± 0.38	6.22	6.31 ± 0.36	5.71
Taste (1–7)^a^	0.3521	6.27 ± 0.39	6.22	6.35 ± 0.43	6.77	6.41 ± 0.38	5.93
Aftertaste (1–7)^a^	0.7946	1.19 ± 0.23	19.33	1.24 ± 0.26	20.97	1.16 ± 0.27	23.28
Overall acceptability (1–7)^a^	0.8546	6.24 ± 0.32	5.13	6.14 ± 0.33	5.37	6.21 ± 0.34	5.48

a *Higher values indicate greater preference*.

*$\bar{X}$ = mean value*.

*σ = standard deviation*.

*CV = coefficient of variation*.

## Discussion

The use of human food flavorings that are aversive to wild birds was tested to reduce feather pecking in caged laying hens under commercial conditions. We monitored behavior, plumage condition, performance, and egg quality parameters of hens aged from 26 to 76 wk and discuss our results in the following sections.

### Behavior

Spraying hens' feathers with two DMA-based repellents had no effect on hen behavior, including feather pecking. Repellent application tended to affect only one observed behavior, namely, cage pecking. Looking at cage pecking it was performed more by control birds than by the birds exposed to the repellent T spraying. The reason for this remains unclear. It should be noted that most chicken-repellent studies have not simulated commercial conditions in terms of production-cycle duration, housing system, genetic strain used, and method of repellent application. Hence, previous approaches and techniques cannot be used to draw direct conclusions about the effects of different repellents in commercial conditions. Therefore, our research is the first attempt to conduct a repellent study in a commercial production setting while taking basic principles of scientific inquiry into account. Kare (18) reported the successful reduction of injurious feather pecking in laying hens using DMA-based repellents. Following his recommendation, in our study the DMA concentration in repellents P and T was 0.78% (vol/vol) and 4.50% (vol/vol), respectively. At least in the case of repellent T the concentration was well above the recommended concentration of ≤ 1%, which has proven to be effective (aversive) on every species of bird tested in the past (16, 24, 25). Our result is thus unexpected. However, the way birds respond to chemical repellents depends on several factors, e.g., target species, active chemical substances in the repellent, route of administration, formulation, mode of action, environment in which the particular repellent is used (26) and materials to be protected (27). This makes it very difficult to compare our results with those of previous studies where DMA-based repellents were used to deter birds from causing damage to grapes, fruits, cereals, grass on pastures (28, 29) and to discourage different species of wild birds (e.g., starlings, red-winged blackbirds, pigeons, jungle fowl, herring gulls, ring-necked pheasants, mallard ducks, Canada geese) from causing losses in livestock feedlots (16, 25). Further, in our study the repellents were homogeneous mixtures of two to three active substances (DMA, MPA, geraniol), while in other studies, the repellents contained only DMA as an active substance. In the case of mixtures, possible interactions between the active substances and/or other components of the mixture (e.g., solvents, adjuvants) should be considered, noting that it is impossible to predict in advance whether the components of the mixture will interact antagonistically, synergistically, or additively (26). Two possible reasons may explain why we did not observe any repellent effect on the hens' behavior: either the repellents did not affect the behavior at all or their effect faded away before we started with behavioral observations. The repellents, short-term effectiveness could be explained by the fact that DMA, as the main active agent, is a highly volatile substance (30) and the addition of solvents (repellent T contained the solvent propylene glycol and repellent P the emulsifying agent Tween 80) further increases its volatility. In addition, DMA is highly sensitive to photodegradation when exposed to incandescent, fluorescent, and ultraviolet light sources (31). Substantivity, a parameter indicating the persistence of the effect (lasting property) of a substance after its application, might also be short as individual values of the three active substances included in our repellent mixtures are 48, 56, and 60 h at 100% for DMA, MPA, and geraniol, respectively (32). With regard to the persistence of repellents, the possibility of being absorbed into the feathers should also not be overlooked (33). Our results may imply the repellent mixtures we used acted as primary chemical repellents. Primary repellents, to which DMA also belong (34), are congenitally aversive to birds, meaning that avoidance is not learned as in secondary repellents but birds perceive them as noxious. Their aversive response is triggered by the activation of nociceptive receptors in the trigeminal nerve, causing sensory pain (24). Consequently, avoidance occurs immediately as the stimulus is perceived, i.e., prior to ingestion, and in a relatively short time gradually disappears when the stimulus is removed (25). The latter might also have happened in our study. Results of the present study are consistent with the results of Harlander-Matauschek and Rodenburg (15), who compared the repellent effect of quinine and other bitter natural products such as garlic, almond oil, clove oil, and clove in laying hens. Quinine, as a secondary repellent, had a strong and long lasting effect, while other tested substances had a shorter effect, most likely because they are primary repellents. It is known, that in secondary repellents the avoidance response is longer and persists even in the absence of the malaise-producing stimulus (35). Dieter et al. (36) also recorded no long lasting effect of different commercially available bird repellents containing methyl anthranilate (MA) in deterring geese from crop damage. MA is a primary repellent (36, 37) which has proven to activate the same nociceptive receptor (TRPA1) as DMA (38) and is as effective as DMA (24). In our study, the repellent mixtures might have been effective in reducing severe feather pecking if the hens had been able to pair them with another sensory cue, e.g., color. Burne and Rogers (39) in their study of passive avoidance in domestic chicks reported that chicks trained to peck a red bead coated with MA 10 min after training pecked less the red bead which was not coated with MA, suggesting they had associated the red bead with MA. Severe feather pecking resulting in plumage damage was observed in all laying hen housing systems (40) in up to 86% of flocks (6). If spraying laying hens with DMA-based repellents had proven to be effective for reducing feather pecking, such a management procedure should serve more as a curative measure during feather pecking outbreaks than as a method for controlling feather pecking (15), such as beak trimming and/or reduced light intensity. Since feather pecking is such a vast and serious problem, the goal should not only be to cure the consequences but to try to solve the underlying mechanisms causing this injurious behavior. Although some progress in preventing feather pecking has already been made in the fields of genetics and management, it remains a complex, multifactorial, and unresolved problem [e.g., (6, 41, 42)] that still calls for the application of not only preventive but also curative measures.

### Plumage Condition

Despite the fact that applying repellents P and T failed to reduce feather pecking, plumage damage, when taking all six body parts together into account, was higher in the repellent P and T treatments than in the hens in the control treatment. Control hens had a better plumage condition in their wings and back areas. More feather damage in the cloaca/vent and breast area was observed in treatment T compared to treatments P and the control. In general, the feathers that came into contact with the repellents were fairly physically damaged. They were dry, rigid, fragile, and broken (personal observation). An explanation is that one or more substances in repellents T and P may have had a negative effect on feather condition. It is known that both DMA and MA are phytotoxic (30, 43). When applied to plants, their drying or desiccating properties become visible, resulting in serious damage to both the leaves and fruit. Although the structure of leaves or fruit is completely different from that of feathers, it may be that DMA affects certain physicochemical properties of hen feathers as well. Chicken feathers contain ~91% protein called keratin, 1% lipids, and 8% water (44). Feather keratin is highly crosslinked with cysteine linkages, which give strength and stiffness while protecting it from degradation. However, when keratin is exposed to alkaline solutions, reducing agents, or oxidizing agents its structure is disrupted (45). The degradation of keratin can also be achieved by keratinases, proteolytic enzymes that may cleave the protein chain of keratin at many sites. Keratinases are produced by feather-degrading bacteria that are found on the feathers of chickens as well as on the feathers of wild birds (46). Although birds have certain defense mechanisms against feather-degrading bacteria, one of them being the uropygial (preen) gland secretion (47), the findings of Kent and Burtt (48) show that these bacteria have a negative effect on feather condition. The question of whether repellent chemicals stimulated the growth of feather-degrading bacteria and thus the keratinolytic activity and condition of the feathers remains unsettled. With increasing hen age, plumage condition became worse regardless of the treatment, which corresponds with the findings of previous studies (49–51).

### Performance

Analysis of mean feed intake showed differences between test periods, however, no regular trends in feed intake were observed with hen age. The application of repellents P and T reduced plumage condition and increased feed intake, albeit only numerically. Such an association is expected since feathers protect the body from heat loss (52) and, when excessive feather loss occurs, a hen consumes more feed to compensate for the heat lost from the denuded areas (53) and feed costs rise. Preventing excessive feather loss therefore can have an important impact on flock health and profitability.

### Physical and Sensory Properties of the Eggs

The comparison of the treatments showed that three correlated traits, namely albumen pH, albumen height, and Haugh units, were inferior in the T group compared to the control and P group. Albumen height or height of the inner thick albumen and Haugh units are the measures most often used to quantify the viscosity of the thick albumen. Another important indicator of albumen quality is its pH value (54). Some studies show that the ingestion of certain compounds or contaminants (e.g., crude oil, vanadium) (55, 56) may reduce albumen quality. It is possible that the ingestion of repellent T led to lower albumen quality. Another reason that could explain the lower albumen quality in the repellent T treatment is that this repellent, through ingestion or absorption via the skin, influences the interaction between individual components of the albumen that cause it to thin. Since the viscosity of the egg albumen significantly determines some of its functional properties, especially the emulsifying properties and the shelf life of eggs, the effect of repellent T in this respect is undesirable. On the other hand, higher albumen pH values make it easier to peel hard-boiled eggs (57). Changes in the physical characteristics of the eggs that occurred over time, i.e., with the aging of the hens, were predictable and in line with the findings of other researchers [e.g., (58–60)]. In order to determine whether any odor absorption as related to repellent treatments had been imparted to the eggs, a descriptive sensory test was used. When the panelists' scores were compared, no repellent treatment effect on the final sensory components of the raw and hard-boiled eggs was found. Two plausible explanations may be offered for the absence of foreign odors in the eggs. One is that the eggs and chemicals in the repellents did not come into direct contact while the second is that, due to being short-lasting, the odor of the repellents was no longer detectable at the time of the egg sampling.

## Conclusions and Applications

The present study was designed to evaluate the aversive potential of relatively inexpensive and ecologically acceptable mixtures of substances (DMA, MPA, geraniol) for controlling feather pecking in the commercial laying hen production system. No evidence was found that spraying hens' feathers with DMA-based repellents plays a significant role in reducing feather pecking behavior. There are two possible explanations for this: either the repellent mixtures had no effect on chicken behavior at all or they acted as primary repellents and lost their efficacy in a relatively short time. However, the physical structure of the feathers was degraded in the repellent treatments. Moreover, egg quality, defined by parameters like albumen height, Haugh units, and albumen pH, was lower in the repellent treatments than in the control treatment. We therefore do not recommend the use of these repellents as a management practice to prevent/limit feather pecking behavior in commercial farm conditions.

## Data Availability Statement

The datasets generated during the current study are available from the authors upon request.

## Ethics Statement

The research was reviewed and approved by the University's Council for the Protection of Animals Used in Experiments and at the state level proceeded under permit number U34401-26/2017/10. All three repellent substances employed in this study are currently authorized for use as flavors in food (Regulation EU 873/2012) and, according to evaluations of the Joint FAO/WHO Expert Committee on Food Additives (JECFA) have no harmful effects on human and animal health or on the environment (61). Further, the other two substances used, namely polysorbate 80 (included as an emulsifier) and propylene glycol (serving as the solvent) are recognized by the JECFA as safe for use in food.

## Author Contributions

DT, DJ, and MZŠ conceived, designed, and directed the study. MZŠ, DJ, and MP performed the measurements. DT and MZŠ derived the statistical models and analyzed the data. DT and DJ designed the figures. DT wrote the manuscript with the support of MZŠ and DJ. Ideas and comments from all authors helped shape the research and manuscript.

## Conflict of Interest

The authors declare that the research was conducted in the absence of any commercial or financial relationships that could be construed as a potential conflict of interest.
